# Protocol for the SELECT study: a sequential mixed methods study of the selection of UK medical students into clinical academic training

**DOI:** 10.1186/s12909-024-06065-y

**Published:** 2024-10-07

**Authors:** Matthew H. V. Byrne, Claudia Chan, Akamiya Karas, Eabha Lynn, Catherine Dominic, Robert Bain, Jonathan C. M. Wan, Andrew D. Clelland, Siena Hayes, Aqua Asif, Anna Harvey Bluemel, Jasper Mogg, Leigh Lawrence, Helen Church, Gabrielle Finn, Megan E. L. Brown

**Affiliations:** 1 Nuffield Department of Surgical Sciences, University of Oxford, Churchill Hospital, Old Rd, Headington, Oxford, OX3 7LE UK; 2 School of Medicine, University of Dundee, Ninewells Hospital, Dundee, UK; 3 Manchester University NHS Foundation Trust, Manchester, UK; 4 Lancaster Medical School, Lancaster, UK; 5 Norwich Medical School, University of East Anglia, Norwich, UK; 6 School of Medicine, Newcastle University, Newcastle upon Tyne, UK; 7 University College London Hospital, London, UK; 8 Princess Alexandra Hospital, Harlow, UK; 9 Kings College Hospital, London, UK; 10 Division of Surgery and Interventional Science, University College London, London, UK; 11 Gateshead Health NHS Foundation Trust, Gateshead, UK; 12 London, UK; 13 School of Medicine, University of Nottingham, Nottingham, UK; 14 University of Manchester, Manchester, UK

**Keywords:** Academic selection, Intern selection, Resident selection, FY1, SFP, AFP, Mixed methods, Document analysis, Survey

## Abstract

**Background:**

Internationally, there has been a move towards fostering diverse healthcare workforces that are representative of the patient populations they serve. Selection criteria for academic-clinicians often aim to capture skills and attributes that demonstrate both clinical and academic excellence. Currently, it is not known whether the selection criteria for early academic-clinical careers advantage or disadvantage certain ethnic or socioeconomic groups. The UK has a structured route of integrated clinical academic training with entry level training for newly qualified doctors administered through the ‘Specialised Foundation Programme’ which provides protected time for research within the first two years of postgraduate clinical training. In this study, we aim to identify what selection criteria are used within the UK Specialised Foundation Programme, and how these relate to demographic factors.

**Methods:**

We will perform a mixed methods study consisting of a document analysis of person specifications and selection criteria used in the 2024 UK Specialised Foundation Programme, and a national cross-sectional survey of current medical students in the UK. We will obtain the person specifications, selection criteria, white space (open ended questions used during shortlisting) and interview questions and mark schemes from each Specialised Unit of Applications via information available on their websites or through Freedom of Information requests. Our survey will collect information relating to demographic data, selection criteria, and perceptions of specialised foundation programme selection.

**Discussion:**

International literature has demonstrated inequity in academic markers used in selection of post-graduate clinicians and that disadvantages caused by selection can compound over time. As such it is important to understand what inequity exists within the selection of early academic-clinicians, as this can help inform more equitable selection practices and help nurture a more diverse academic-clinical workforce.

## Introduction

Internationally, there has been a shift towards fostering diverse workforces that are representative of the patient populations they serve [1–3]. As well as promoting a more inclusive work environment through this agenda, health outcomes are enhanced when there is a workforce of greater diversity [4, 5]. Moreover, a diverse academic workforce helps broaden perspectives, increases innovation, and improves uptake of interventions [6].

Clinical academics play a vital role in advancing medical practice and improving patient care through research [7]. They aim to bridge the gap between clinical practice and academic inquiries, translating scientific findings to real-world practice and refining evidence-based medicine, with the goal to address ongoing healthcare needs and enhance patient outcomes.

In the UK following the completion of the medical degree, medical students apply to foundation training – a two year period of training following graduation covering a range of placements in both primary and secondary care. Medical students can also apply to a specialised foundation programme (SFP), previously known as the academic foundation programme, which has time dedicated to academic, education, or leadership research or study. The SFP allows foundation doctors to complete core competencies of the foundation programme alongside experiences conducive to academia integrated into the two-year training pathway. The purpose of the SFP is to allow sufficient preparation for doctors who are interested in further academic training and doctoral degrees subsequent to the foundation programme.

In order to develop a competent and diverse medical workforce, rigorous selection criteria are needed to ensure alignment of skills, and interest, with role requirements. This is particularly important for academic-physicians selection, which needs to capture skills, attributes and motivations that demonstrate both clinical and academic excellence. Academic achievements can be predictors of success in future clinical and academic practice [8–10], and therefore academic-physician selection often incorporates markers of academic merit, such as presentations, publications, and prizes.

Yet, strictly academic criteria might represent a limited view of an individual's achievements. For example, individuals who excel in sports may exhibit numerous attributes that could be applied to an academic career such as determination, focus, and disciplined practice [11]. Extracurricular activities have also been associated with better academic performance at medical school [12].

As a result, some criteria for both clinical and academic selection include extracurricular activities such as sports achievements, and graded music proficiency (NHS England, 2023b) [13]. American medical schools use a holistic admission process, valuing various experiences and qualities beyond academic achievements alone to diversify the medical workforce [14, 15]. This approach values not only academic metrics but also qualities such as resilience, empathy, cultural competence, and adaptability displayed through various means including personal essays, letters of recommendation, community exposure, and extracurricular activities.

While extracurricular achievements are impressive, there is a risk that by including them in selection criteria they may disadvantage some students. For example, a student whose university experience is entirely funded by family, student loans or other personal funds may have more opportunities to undertake extra curricular activities than a student who has to work to fund their degree, has caring responsibilities, or who has a disability. In the UK, it was highlighted that students from a higher socio-economic background were 20% more inclined to engage in sporting and music extracurricular activities compared to their peers from a lower socio-economic background [16]. This may also compound existing differential attainment in these groups, as academic performance at medical school is worse for those who need to work or have caring responsibilities alongside their degree [12].

We know some selection criteria for SFP have been challenged legally because they might indirectly discriminate against certain groups [17]. For example, one programme produced selection criteria including graded music proficiency [18] - a qualification thought to be more accessible to students from higher socio-economic classes, due to the private tuition and exam fees required.

Indirect discrimination occurs when processes apply to individuals equally but disadvantage some groups more than others - for example based on age, disability, race, religion or belief, sex, pregnancy and maternity, sexual orientation, gender reassignment, and marriage and civil partnership. In the UK, these characteristics are protected by legislation [19], with similar laws in several countries [20–24]. Moreover, the United Nations describes how discrimination based on socioeconomic status is a systemic issue and that “States should ensure their anti-discrimination framework effectively prohibits (direct and indirect) discrimination on grounds of socio-economic disadvantage” [25]. Discriminatory practices not only undermine the principles of equity and fairness but also inhibit the potential for healthcare and academic settings to benefit from diverse perspectives and experiences.

Discriminatory practices in selection of health and academic professionals is a longstanding issue and limits the diversity within the workforce [26]. For example, implicit bias influences medical school admissions and contributes to the underrepresentation of minority groups [27]. Several studies have emphasised structural barriers, such as racial and socioeconomic factors that lead to underrepresentation in medical school admission [28–31]. Such inequities result in differential access to resources for application preparation and extracurricular opportunities, which are valued in the medical school admissions process. Academic awards and honours which are used as a marker of academic excellence during medical school are awarded less often to minority groups [32–36]. The problem of underrepresentation continues after medical school where there is reduced diversity in postgraduate training programmes and academic careers [37–39]. Despite the increasingly diverse population and medical graduates, there remains a lack of representation in specialty training programmes [40–42].

By including criteria that could indirectly discriminate against certain groups of individuals, there is a risk that diversity within academic medicine is limited. Capers argues that senior leaders within healthcare institutions hold a vital role in facilitating diversity within the workforce [43]. This is especially important for those who hold positions on selection committees, whom Capers charges with the responsibility of ensuring diversity statements guide the questions they use [43].

Currently, it is not known whether the selection criteria for early academic-clinical careers advantage or disadvantage certain groups. In this study, we aim to identify what selection criteria are used within the UK specialised foundation programme, and how these relate to demographic factors. We hypothesise that extra-curricular types of selection criteria disadvantage certain groups and do not align with the overarching aims of fostering diversity within academic programmes.

## Methods

We will perform a sequential mixed methods study consisting of a document analysis, followed by a cross-sectional survey of medical students in the UK. We will follow the CARDA guide for document analysis [44] and the STROBE guideline for cross-sectional studies [45].

We will perform a document analysis on the person specifications, selection criteria, white space and interview questions and mark schemes used within the UK specialised foundation programme. This will provide a comprehensive overview of early career clinical-academic selection. We will then use a survey, whose questions will be informed by the selection criteria as well as a review of the literature, to provide data on how these criteria relate to demographic information such as socioeconomic status, ethnicity, and disability. This will provide data on whether different selection criteria used advantage or disadvantage certain groups of students.

### Document analysis

Specialised Units of Applications were identified from the UKFPO website in September 2023 [46].

We will obtain the person specifications, selection criteria, white space and interview questions and mark schemes from each Specialised Units of Application website. Where these are not available we will ask the Specialised Units of Applications to provide it via email through a freedom of information act request (Table 1), using an approach we have previously described [47]. Our request was reviewed by an expert in evaluation to check that questions were appropriate. We staggered our initial requests so that we received and reviewed five responses before wider dissemination to ensure questions and responses are appropriate. Repeat FOI requests will be used to clarify any unclear responses.

**Table 1:** FOI request to Specialised Units of Applications. Questions where information has already been obtained from the website will be omitted

Dear [Recipient's Title and Name, Sir or Madam if unknown], My name is [Name] I am a medical education researcher conducting a study on the criteria used for the academic selection of final year medical students in Specialised Foundation Programmes. Under the provisions of the Freedom of Information Act, I am seeking information regarding the 2024 Specialised Foundation Programme application cycle for the Specialised Unit of Application: [Specialised Unit of Application name]. I kindly request the following information: • What are the person specifications used when shortlisting applicants for your Specialised Foundation Programme positions? (By person specifications we are referring to an outline of the essential and desirable attributes of candidates) • What selection criteria do you use when shortlisting applicants for your Specialised Foundation Programme positions? (By selection criteria we are referring to the criteria used by the committee to shortlist candidates). Please provide the score available for each criterion and the total score available for each section. For example: ‘Publications: 1 point per PubMed indexed publication; total 5 points available’. • Do you use white space questions to shortlist candidates? And if yes, please provide the questions and marking proforma that you use. • What is the relative weighting of the criteria and white space questions when shortlisting applicants? For example, criteria and white space questions are weighted equally, or 20% for criteria and 80% for white space questions. • Do you use an interview following shortlisting of candidates? And if yes, please provide the questions and marking proforma that you use. • Do interview candidates perform an appraisal of evidence (e.g. a manuscript) as part of the interview? Please answer Yes or No. • What is the relative weighting of the shortlisting score and the interview score? For example, the candidate is selected on the interview score alone, or 20% for shortlisting score and 80% for interview score. • How many applications do you receive to your Specialised Unit of Application in total? • Do you subscribe to the Disability Confident Scheme, where interviews are offered to anyone with a disability whose application meets the minimum criteria for the post? Please answer Yes or No. • Have any candidates been interviewed via the Disability Confident Scheme? Please answer Yes or No. I kindly request that you provide this information in the form of the documents you use to shortlist candidates. In the event that these documents are not available, please provide the answers to each question as a text response. If your Specialised Unit of Application covers multiple regions, foundation schools, or universities, and the person specifications, selection criteria, white space questions, or weighting differ between these locations, I kindly request separate responses for each distinct location. If any clarification is needed or if further information is required, please contact me at mededcollaborative@gmail.com. I appreciate your time and cooperation in providing this information, which will contribute significantly to my study. Thank you in advance for your assistance.	

To summarise each criteria we will perform a content analysis, code each item, and present count and percentages for each code. Where quantitative analysis is not possible we will perform a descriptive analysis.

### Survey development

We will develop our survey following the AMEE guide on developing surveys [48]. To develop our questionnaire, first we will review the current selection criteria and person specifications used for specialised foundation programmes in the UK. Second, we will review the literature for similar studies or other selection criteria to identify additional questions. Demographic data will include protected characteristics: gender, sex, age, ethnicity, pregnancy, religion, sexual orientation, marriage status, disability [19]; and non-protected characteristics including markers of socioeconomic status (recipient of free school meals, attendance of fee-paying school as a child, index of multiple deprivation, and parental occupation), caring responsibilities, long term unplanned absences from medical school, and international student status. After we develop a preliminary list of questions, these will be refined through discussions as a group.

Based on our previous experience of analysing criteria used to determine the award of honours awarded at graduation by medical schools [47], we anticipate that there will be considerable heterogeneity in the selection criteria used within the UK Specialised Foundation Programme. If this is the case, we will not be able to capture every single selection criteria used for our target population as the survey will be too long. Instead, we will aim to limit the number of questions within each broad domain (e.g. presentations, publications), where possible we will aim for only one question per domain. This will help limit the length of the survey, and reduce survey fatigue and participant dropout, while being broadly representative of the questions used [48].

We also anticipate that white space and interview questions will be challenging to integrate into a succinct survey - as these might result in too many free text responses. Therefore, we anticipate that our survey will be formed primarily of quantitative questions from the selection criteria rather than white space and interview questions.

From our experience using FOI requests before, there can also be long delays in responses and clarification [47]. Therefore, we will proceed with survey development once we have reached ‘data saturation’ - the point where we believe we have identified all broad domains of criteria [49].

#### Expert validation, cognitive interviews, and pilot study

Once we have finalised our survey we will review it with 1-2 experts in evaluation for expert validation. We will perform 5-10 cognitive interviews [50], and a pilot study of 20-30 final year medical students to improve face, content, and response process validity [48, 51]. We will discuss the results of our expert validation, cognitive interviews, and our pilot study as a group and identify whether any questions should be altered or removed.

### Sample selection

#### Inclusion criteria

All medical students studying at a U.K. medical school (we will perform a sub-group analysis for penultimate and final year medical students). Medical schools will be identified from the U.K. Medical Schools Council website. We chose to sample all medical students so that if there are differences in demographics we can identify which year in medical school these occurred.

#### Sample size

We will aim to recruit 1000 students in total. This sample size has previously allowed us to perform detailed analysis of demographic information against multiple factors [52].

### Survey dissemination

We will contact all eligible UK medical schools via their general enquiries email address and ask them to distribute the survey. We will use the MedEd collaborative network to disseminate the survey locally. We will post once per week on social media platforms and share the link to the survey.

#### Collaborative authorship

We will aim to recruit 1-5 collaborators per university, to be listed as a collaborative author on the outputs of our survey, penultimate and final year collaborators are required to recruit 30 responses from either penultimate or final year students at their medical school combined. If collaborator(s) from lower years are recruited, this will be 30 responses from their year. Collaborators are also required to participate in the pilot study and provide feedback (20-30 collaborators) and participant in a ‘cognitive interview’ (5-10 collaborators) if requested. These individuals will be listed as collaborators under the group authorship: ‘MedEd Collaborative’.

Collaborators will be recruited from existing Medical, Medical Education, Surgical, and Academic societies within each university first, where this is not possible we will then recruit any remaining collaborators via the @MedEdCollab social media.

#### Survey analysis plan

To study whether selection methods of Specialised Foundation Programmes either advantage or disadvantage certain demographic groups, we will assess how certain demographic criteria are associated with scores determined by different selection metrics.

First, Generalised Linear Models (GLM) will be used to assess the impact of demographic factors on selection criteria and outcomes. Logistic regression will be used to predict the likelihood of selection criteria and outcomes based on these factors. The weights of such models may provide insights into disparities between demographic groups. Separately, chi-square tests for independence and multivariate analyses of variance (MANOVA) may also be utilised to explore whether associations exist between demographic variables and selection criteria and outcomes. Where multiple testing is performed, multiplicity correction would be performed using the Benjamini-Hochberg method.

For qualitative responses, we will perform a thematic analysis using Braun and Clarke’s reflexive approach [53, 54].

### Ethical approval

The document analysis was reviewed by the University of Oxford Medical Sciences Interdivisional Research Ethics Committee, and the document analysis did not require ethical approval (reference: R92692/RE001). Once our survey has been finalised we will seek ethical approval prior to distribution.

### Expected outputs

We will aim to publish our data in peer reviewed medical or scientific journals, and submit our project to national and international conferences.

## Discussion

This paper aims to identify the selection criteria used within the UK specialised foundation programme, and how these relate to demographic factors. This study addresses a gap in the literature regarding a lack of research surrounding early academic-clinician selection both within the UK and globally, and the demographic factors that may influence success in obtaining these positions.

While early academic-clinician selection has not been widely studied, international literature has demonstrated inequity in academic markers used in selection of post-graduate clinicians. Most scholarship on this topic originates in the USA, where Alpha Omega Alpha society membership (an honours society for USA medical graduates; medical schools can elect up to 16% of their graduates to join) [32], and honours during clerkships have been long established predictors of career success [55] - including successful application to residence programmes [56], and academic employment [57]. More recently a study of US Medical Graduates seeking general surgical resident posts found that Alpha Omega Alpha Honors Society membership, attending ‘top’ medical schools, research experience and higher USMLE scores were the most important factors in securing a position [58]. Consequently, this can promote worsening differential attainment. Gender disparities have been observed in the awarding of honours in some programmes [59]. Medical students of minority ethnicities [60], as well as students from lower socio-economic backgrounds [61], who are less likely to be members of Alpha Omega Alpha, and less likely to receive Honours. We also know that, in the UK, students from socioeconomic disadvantaged backgrounds or minority ethnicities can perform worse on average during their medical school education and postgraduate career progression due to inequitable practices within medical education [62, 63]. Early disadvantages are particularly important, as these can compound overtime [64]. Indeed, UK students who entered medicine through ‘gateway programmes,’ designed to promote access to the profession for those from otherwise underrepresented backgrounds, are known to be less likely to pass postgraduate college membership examinations on their first attempt, and less likely to gain postgraduate training positions on their first application [65].

In February 2024, the UKFPO announced that from 2025 entry onwards, the specialised foundation programmes would be brought into the same pool as other foundation programmes, and applicants assigned to programmes based upon a new system of “preference informed allocation” - where students are assigned by an algorithm based on their preference rather than any selection criteria [66]. According to the Northern Ireland Medical and Dental Training Agency, part of the rationale for this change was “in response to concerns raised across the UK that the use of interviews and white space answers is problematic on equity, fairness, diversity and differential attainment grounds” [67]. In response, leading clinical academics in the UK, including 13 specialised foundation school leads representing 11 specialised units of application, co-signed a letter to the Lancet journal expressing their own concerns that these changes will disincentivize medical students from a clinical academic career at a time when the UK has a shortage of clinical academics [68]. In this letter, Barratt et al. referenced data presented by the General Medical Council at the Clinical Academic Training Forum 2022 meeting which supported that the protected characteristics of Specialised Foundation Programme doctors were representative of UK medical school applicants. Barratt et al. recommended tailored ‘levelling up’ approaches rather than a blanket change to preference-informed allocation [68]. This study will, in part, examine the validity of these concerns from both parties by examining what criteria are used in the selection process. However, further study is needed to ascertain whether preference informed allocation will appropriately mitigate or exacerbate potential inequities in access to clinical academia in the UK.

We hope this study will distinguish which selection criteria advantage or disadvantage certain groups of students from questions that are not associated with demographic factors, and potentially identify groups of students who might need additional support. Identification of these issues can allow targeted intervention and positive changes towards more representative and equitable selection processes in medical training. This could allow for a more equitable set of selection criteria that can be further investigated for use to select candidates who are best prepared for the challenges of a clinical-academic career. This is important for both academic and non-academic clinician selection internationally, as many of these selection criteria are used in post-graduate academic and specialty application selection in the UK and internationally [13, 56, 58, 69–72].

### Strengths and limitations

The use of a sequential mixed methods approach will provide a comprehensive overview of early academic-clinician selection, which can then be investigated through a survey of students with detailed descriptions of demographics, encompassing both those protected by the Equality Act 2010 [19], socioeconomic status [25], and other relevant demographic factors we identify through the literature such as carer status and whether participants worked during their studies [12].

We chose to use a survey as this allowed us to identify the relationship between demographic factors and selection criteria. Alternatively, we could have used interviews, however, interviews would not have allowed us to quantify the relationship between many selection criteria and demographic factors through quantitative analysis such as multiple regression. We could have reviewed a medical education database such as UKMED. While these are more representative, as all students are included, these databases do not capture all selection criteria used.

We will use a convenience sampling approach, so there is a risk of self-selection bias. We will aim to mitigate this through the use of anonymous reporting, and disseminating our survey via a variety of routes. This is a cross-sectional survey, and as such, we cannot perform a longitudinal analysis. As such, we will not be able to identify which selection criteria are associated with later academic success. Ideally this would be examined by performing an analysis of a medical education database that can track career progress, such as UKMED. However, these databases do not capture all the selection criteria used. Therefore, an area for future study may be a survey of successful early career academic-clinicians (for example those who go onto obtain doctoral fellowships) to collect retrospective data from a similar timepoint (e.g. at the end of their final year of medical school) using the same selection criteria, to see whether any selection criteria are a best predictors of future success. This study will be unable to understand why selection committees developed the criteria they used, and this may be an area for future, interview-based study. These limitations are important to understand so that the best candidates can be appointed using an approach that is fair – an important requirement for funders of these positions.

## Data Availability

Not applicable.

## Data availability

No datasets were generated or analysed during the current study.
